# Near-Complete Genome Sequence of Ryegrass Mottle Virus from Irrigation Water in Ecuador

**DOI:** 10.1128/MRA.00037-21

**Published:** 2021-05-06

**Authors:** Fiama Guevara, Francisco Flores

**Affiliations:** a Centro de Estudios de Posgrado de la Universidad de las Fuerzas Armadas ESPE, Sangolquí, Ecuador; b Departamento de Ciencias de la Vida y Agricultura, Universidad de las Fuerzas Armadas ESPE, Sangolquí, Ecuador; c Centro de Investigación de Alimentos, Facultad de Ciencias de la Ingeniería e Industrias, Universidad UTE, Quito, Ecuador; Portland State University

## Abstract

In this work, we report the near-complete genome sequence of ryegrass mottle virus identified in irrigation water through next-generation sequencing and *de novo* assembly. The genome is 4,247 bp long, arranged in five open reading frames with a 5′ untranslated region (UTR) of 87 nucleotides and a 3′ UTR of 247 nucleotides.

## Abstract (precis)

In this work, we report the near-complete genome sequence of Ryegrass mottle virus identified in irrigation water through next-generation sequencing and *de novo* assembly. The genome is 4,247 bp long, arranged in five open reading frames with a 5′ untranslated region (UTR) of 87 nucleotides and a 3′ UTR of 247 nucleotides.

## ANNOUNCEMENT

The ryegrass mottle virus (RGMoV) is a single-stranded, positive-sense RNA virus belonging to the *Sobemovirus* genus and *Solemoviridae* family (1). RGMoV was first isolated from Italian ryegrass (Lolium multiflorum) and cocksfoot (Dactylis glomerata), but this virus can readily infect wheat (Triticum aestivum), barley (Hordeum vulgare), and oat (Avena sativa), causing mottling and necrotic symptoms on leaves (2).

Viruses can survive in different environments and can be transmitted by different vectors that allow them to spread effectively (3, 4). Water plays an important role in the dissemination of highly stable pathogens, which can become a problem for human and ecosystem health. Viruses can be resistant to standard water treatment processes, posing a risk for the use of reclaimed water for agriculture or industry or as a nonpotable public water supply (5).

Plant viruses known to be resistant and transmitted by water are members of the *Tobamovirus* genus and *Tombusviridae* family (5). Sobemoviruses are transmitted by insect vectors, by soil, and through mechanical inoculation (6). Although there are no available data about waterborne transmission of RGMoV, it has been shown that rice yellow mottle virus, another species of sobemovirus, can be transmitted by irrigation water contaminated with infected guttation fluid (6).

In this work, a metagenomic approach was used to examine plant viruses present in irrigation water. Ten-liter samples were collected in sterile plastic carboys from an irrigation water reservoir in the Tumbaco Experimental Farm of the National Institute of Agricultural Research (INIAP), located in the province of Pichincha, Ecuador. Samples were prefiltered using a 50-μm filter and concentrated by a skim milk organic flocculation method (7). Total RNA was extracted using the SV total RNA isolation system kit (Promega Corp.) according to the manufacturer’s instructions.

RNA libraries were prepared using the Ribo-Zero rRNA removal kit and the TruSeq RNA sample preparation kit v2 (Illumina, Inc., USA) and sequenced with an Illumina NovaSeq 6000 sequencer at Macrogen, Inc. (Seoul, South Korea), generating a total of 63,909,998 reads. All tools were run with default parameters unless otherwise specified. The sequences were trimmed and filtered using Trimmomatic v0.39 (8), and then duplicated sequences were removed using the dedupe tool in BBMap v38.86 (9). Reads were reduced to 32,743,168 reads between 50 and 101 bp long and were assembled using metaSPAdes v3.14.1 software (10), generating a total of 304,518 nonredundant contigs between 80 and 155,245 bp long.

The assembled contigs were compared to the GenBank virus reference database using the BLASTn option from BLAST-2.10.1+ software (11) with an E value of 0.05 and 95% identity threshold parameters. The BLAST search identified six different plant viruses in which a single contig (4,247 bp long, with a GC content of 53.43%) showed sequence similarity to the RGMoV reference genome (12) with 99.72% sequence coverage, indicating that the near-complete genome was assembled. Mapping of deduplicated reads to the RGMoV assembled contig revealed an average coverage depth of 107.681×. Five open reading frames (ORFs) on the positive strand, a 5′ untranslated region (UTR) of 87 nucleotides, and a 3′ UTR of 247 nucleotides were identified on the basis of the reference RGMoV genome, showing its typical organization (13, 14).

More studies are needed to determine the infectivity of irrigation water containing RGMoV and its host range to evaluate its impact on important cultivated crops in Ecuador.

### Data availability.

The near-complete genome sequence of RGMoV strain Ecuador has been deposited in the GenBank database under the accession number MW411579. The raw data are available in the Sequence Read Archive (SRA) under the accession numbers SRR13980363 and SRR13070794.
